# Identification of neural cells activated by mating stimulus in the periaqueductal gray in female rats

**DOI:** 10.3389/fnins.2014.00421

**Published:** 2014-12-18

**Authors:** Shunji Yamada, Mitsuhiro Kawata

**Affiliations:** Department of Anatomy and Neurobiology, Kyoto Prefectural University of MedicineKyoto, Japan

**Keywords:** periaqueductal gray, cFos, mating stimulus, vesicular glutamate transporter 2, FluoroGold, lordosis

## Abstract

Induction of lordosis as typical female sexual behavior in rodents is dependent on a mount stimulus from males and blood levels of estrogen. Periaqueductal gray (PAG) efferent neurons have been suggested to be important for lordosis behavior; however, the neurochemical basis remains to be understood. In this study, we neuroanatomically examined (1) whether PAG neurons activated by mating stimulus project to the medullary reticular formation (MRF), which is also a required area for lordosis; and (2) whether these neurons are glutamatergic. Mating stimulus significantly increased the number of cFos-immunoreactive (ir) neurons in the PAG, particularly in its lateral region. Half of cFos-ir neurons in the lateral PAG were positive for a retrograde tracer (FluoroGold; FG) injected into the MRF. cFos-ir neurons also colocalized with mRNA of *vesicular glutamate transporter 2 (vGLUT2)*, a molecular marker for glutamatergic neurons. Using retrograde tracing and *in situ* hybridization in conjunction with fluorescent microscopy, we also found FG and *vGLUT2* mRNA double-positive neurons in the lateral PAG. These results suggest that glutamatergic neurons in the lateral PAG project to the MRF and are involved in lordosis behavior in female rats.

## Introduction

Lordosis is a typical sexual behavior of a female rodent and is induced by a male mount stimulus under proestrus levels of estrogen. The mount stimulus passes through the anterolateral column of the spinal cord and then inputs into the medullary reticular formation (MRF) and periaqueductal gray (PAG) (Pfaff, 1980). Daniels et al. demonstrated an efferent pathway for lordosis behavior using a pseudorabies virus (PRV) as a transneuronal retrograde tracer (Daniels et al., 1999). When PRV was injected into the lumbar epaxial muscles, which produce a lordosis posture in female rats, the PRV was sequentially labeled in the MRF, PAG, and ventromedial nucleus of the hypothalamus (VMH). The VMH is the main site of action of estrogen for inducing lordosis (Rubin and Barfield, 1983) and estrogen receptor-expressing neurons in this nucleus project to the PAG (Calizo and Flanagan-Cato, 2003). Thus, the PAG and MRF are important relay areas that reflexively change a male mount stimulus into an output for lordosis posture (Pfaff, 1980).

Electrical stimulation of the PAG induces lordosis behavior (Sakuma and Pfaff, 1979a). Conversely, lesions of the PAG (Sakuma and Pfaff, 1979b) or local lesions in the caudal ventrolateral PAG (Lonstein and Stern, 1998) reduce lordosis in female rats. Manual vaginocervical stimulation (VCS), which induces lordosis, is increased cFos expression in the PAG (Pfaus et al., 1996). Neural connections of the PAG to the MRF are involved in induction of an electromyogram (EMG) response in muscles regulating lordosis in female rats (Robbins et al., 1990). These results suggest that PAG efferent neurons activated by a mating stimulus may be related to induction of lordosis, but the neurotransmitter in PAG neurons projecting to the MRF remains to be understood.

Many reports have shown involvement of glutamate and its receptor in lordosis. Intracerebroventricular (icv) administration of N-methyl-D-aspartic-acid (NMDA), an agonist of the glutamate NMDA receptor, facilitated lordosis in ovariectomized (OVX) rats treated with low-dose estrogen (Gargiulo and Donoso, 1995), and activation of lordosis induced by progesterone in estrogen-treated OVX rats was blocked by icv injection of a NMDA antagonist (Gargiulo et al., 1992). A mRNA for *vesicular glutamate transporter 2 (vGLUT2)*, a molecular marker for glutamatergic neurons (Ziegler et al., 2002, 2012), is expressed in the lateral part of the PAG (Oka et al., 2008). Therefore, we hypothesized that the lateral PAG neurons projecting to the MRF are glutamatergic neurons and that these neurons are involved in lordosis. To investigate this hypothesis, we used neuroanatomical methods to examine (1) whether lateral PAG neurons activated by a mating stimulus directly project to the MRF, and (2) whether these neurons are glutamatergic in estrogen-treated OVX rats.

## Materials and methods

### Animals and treatments

Wistar female rats aged 8 weeks were purchased from Shimizu Laboratory Supplies Co. (Kyoto, Japan) and housed under a 12-h reverse light/dark cycle with free access to food and water. After two consecutive estrus cycles, rats were bilaterally ovariectomized and silastic tubing (1.5 mm i.d.; 3.0 mm o.d.; 25 mm length; Dow Corning, Midland, MI) containing crystalline 17 β-estradiol (E2, Nachalai, Osaka, Japan) was implanted subcutaneously under anesthesia with 2–3% isoflurane. We confirmed that the E2 treatment caused hypertrophy of the uterus and induced a high lordosis quotient (>90) against male mount behavior. All experimental procedures were authorized by the Committee for Animal Research, Kyoto Prefectural University of Medicine.

### Sexual stimulation and tissue preparation

One week after OVX and E2 treatment, the rats were assigned randomly to a sexual stimulus condition. Some female rats were placed into a test arena (60 cm long × 30 cm wide × 30 cm high) with a sexually vigorous male (age > 12 weeks) for 1h for mating stimulus at 17:00, and others were placed into the same arena without a male to serve as non-mating stimulated controls. Mating-stimulated female rats received >10 mating stimuli within 15 min. At the conclusion of sexual stimulation, all animals were anesthetized with pentobarbital (Somnopentyl; Kyouritsu Seiyaku, Tokyo, Japan) and perfused with physiological saline followed by 4% paraformaldehyde in 0.05M PB. The brain was immediately removed, postfixed with the same fixative overnight at 4°C, and then kept in 30% sucrose in 0.05M PB at 4°C. Serial coronal sections (30 μm) containing the PAG were obtained using a cryostat (CM 3050 S; Leica, Wetzlar, Germany).

### cFos immunohistochemistry (IHC)

Every fourth section through the PAG (8 sections, from 7.0 to 8.2 mm posterior to the bregma in the brain atlas (Paxinos and Watson, 2006)) from mating-stimulated (*n* = 5) and control (*n* = 5) rats was sequentially incubated with 0.3% H_2_O_2_ in PBS with 0.3% Triton X-100 for 30 min and 2% normal goat serum (NGS) in PBS for 1h at room temperature (RT). Sections were then incubated with primary rabbit antiserum against cFos (1:15,000; Ab-5, Calbiochem, Merck, Tokyo, Japan) for 24h at RT. Immunoreactive (ir) neurons were visualized with a streptavidin-biotin kit (Nichirei, Tokyo, Japan), followed by 3,3′-diaminobenzidine (DAB) with 2.5% nickel chloride, as described our previous method (Takanami et al., 2010).

### Fluorogold (FG) injection into the MRF and FG and cFos double-IHC

Five days after OVX and E2 treatment, rats (*n* = 9) were stereotaxically implanted with a stainless-steel guide cannula (23-gage; Plastics One, Roanoke, VA) in the MRF with the tip end at 11.4 mm posterior and 9.0 mm ventral to the bregma and 0.7 lateral to the midline, according to the brain atlas (Paxinos and Watson, 2006). FluoroGold (FG; Invitrogen, Carlsbad, CA) was dissolved in saline at 2% and unilaterally injected into the MRF at a rate of 0.25 μl/min for 2 min using a microsyringe pump through an internal cannula (26 gage). This procedure was performed under anesthesia with pentobarbital (13 mg/ml Somnopentyl, 0.15ml/100g body weight). Two days after FG injection, some rats (*n* = 6) received sexual stimulation and others (*n* = 3) were used as non-stimulated controls. Brains were processed for FG and cFos double-IHC. After cFos-ir was detected as described above, free-floating sections were sequentially incubated with 0.3% H_2_O_2_ in PBS for 15 min, 2% NGS in PBS for 1 h, and primary rabbit antiserum against FG (1:20,000; Invitrogen) for 24h at RT. FG-ir neurons were visualized with a streptavidin-biotin kit, followed by DAB as a chromogen.

### *vGLUT2 mRNA in situ* hybridization (ISH) and cFos IHC

To detect *vGLUT2* mRNA, cDNA for vGLUT2 (734 bp) was generated by RT-PCR from total RNA of rat hypothalamus. Primers were based on the sequence of rat vGLUT2 (accession number AF271235). The upstream and downstream primers were 5′-CTT CTT GGT GCT TGC AGT GG and 5′-GGA CGA ATG GCC TGA ATG GA, respectively (Ziegler et al., 2002). Non-radioactive free-floating ISH was performed as described previously (Yamada et al., 2007, 2012). Briefly, every fourth section containing the PAG (8 sections, *n* = 6) was acetylated and then hybridized with 2 mg/ml DIG-labeled *vGLUT2* antisense cRNA probes synthesized from cDNA of vGLUT2 using a DIG-labeling kit (Boehringer Mannheim GmbH, Mannheim, Germany) overnight at 55°C. After elimination of excess cRNA probes, the sections were incubated with 1.5% blocking reagent (Boehringer Mannheim) and then with an alkaline phosphatase (AP)-conjugated anti-DIG antibody (1:1000, Roche Diagnostics Corp., Indianapolis, IN) for 2h at 37°C. *vGLUT2*-positive neurons were visualized with a BCIP/NBT solution (1:50, Roche Diagnostics Corp.). After *vGLUT2* ISH, cFos IHC was performed as described above.

### Fluorescent *vGLUT2 mRNA* ISH and FG IHC

Preparation of PAG sections (*n* = 3) after FG injection into the MRF and the procedure until blocking with 1.5% blocking solution is described in the section on ISH for *vGLUT2* mRNA. After blocking, the sections were incubated with a mixture of sheep horseradish peroxidase-conjugated anti-DIG antibody (1:20, Roche Diagnostics Corp.) and rabbit anti-FG antibody (1:1000, Invitrogen) overnight at RT. Then the sections were incubated for 30 min in biotin-conjugated tyramide (1:50 in amplification diluent, PerkinElmer, Waltham, MA). Following several washings, the sections were incubated with a mixture of Alexa 488-conjugated streptavidin and Alexa 546-conjugated anti-rabbit IgG (1:500, Molecular Probes, Eugene, OR) for 2h at RT.

### Analysis and statistics

After staining, the sections were mounted on APS-coated glass slides and covered with a glass micro-cover slip. Non-fluorescent staining was observed under a light microscope (BX 50; Olympus) and photographs of ipsilateral PAG were captured using a CCD camera (DP 21; Olympus). A frame of size of 0.5 × 0.5 mm (region of interest, ROI) was made in the captured lateral PAG and the numbers of cFos-ir, FG-ir, *vGLUT2* mRNA-positive, FG-ir and cFos-ir, and *vGLUT2* mRNA-positive and cFos-ir neurons in the ROI were counted. Immunofluorescent staining was viewed and captured using a LSM510META confocal laser-scanning microscope (Carl Zeiss, Jena, Germany). All values are expressed as means ± SEM. The significance of a difference between mating-stimulated and non-stimulated control rats was evaluated by Student *t*-test.

## Results

### Activation of lateral PAG neurons by mating stimulus

**Figure 1:**
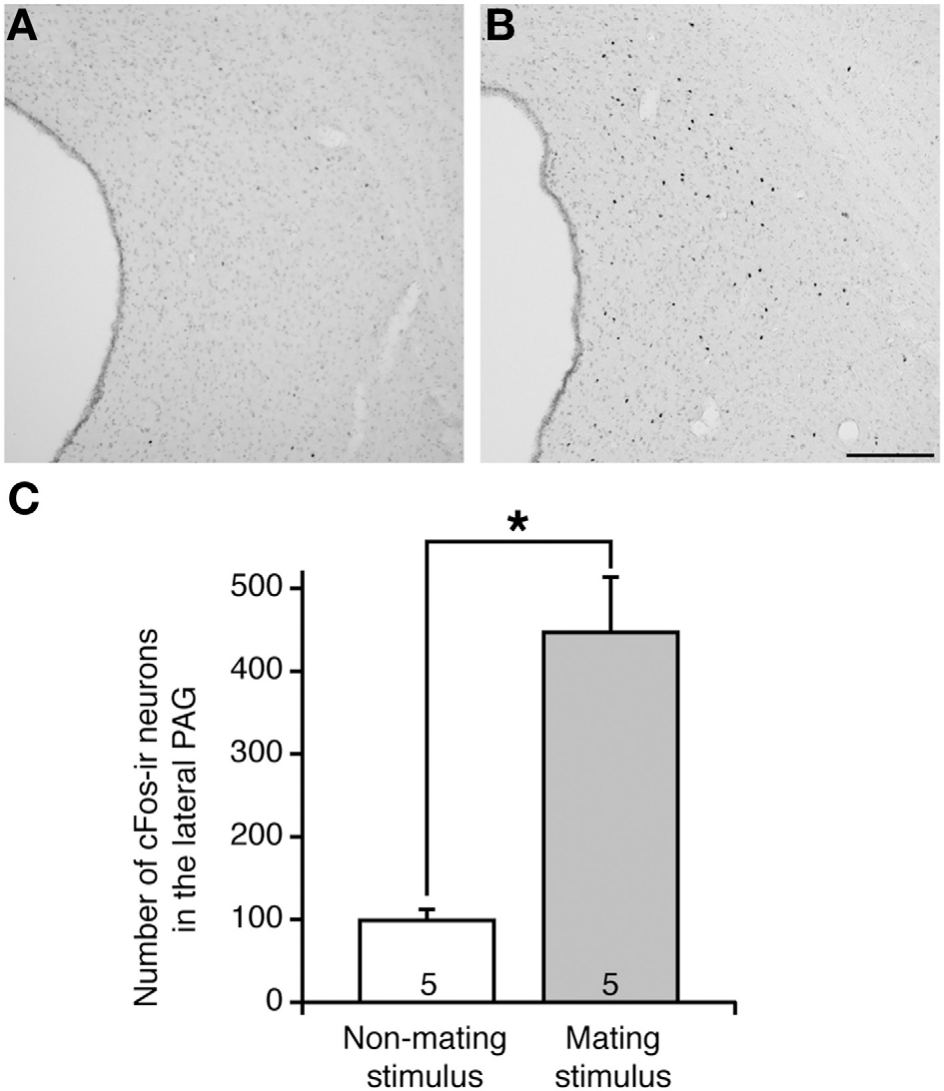
**cFos expression in the lateral PAG in representative female rats following (A) non-mating stimulus and (B) mating stimulus**. cFos-ir neurons are shown by black dots. **(C)** The mean number of cFos-ir neurons in the lateral PAG in OVX+E2 rats after mating stimulus (solid bar) was significantly higher than that in non-stimulated control rats (open bar) (^*^P < 0.05; Student *t*-test). Values are shown as means ± SEM. The numbers in each column indicate the numbers of animals used. Scale bar, 100 μm.

In non-mating stimulated control rats, which were placed in the test arena without male rats, there were few cFos-ir neurons in the PAG (Figure 1A). In contrast, in mating-stimulated rats, many cFos-ir neurons were present in the rostral to caudal parts of the PAG, particularly in the lateral area (Figure 1B). The number of cFos-ir neurons in the lateral PAG in these rats was fourfold greater than that in control rats (*P* < 0.05, Figure 1C).

### Projection of mating-stimulated cFos-expressing neurons in the lateral PAG

**Figure 2:**
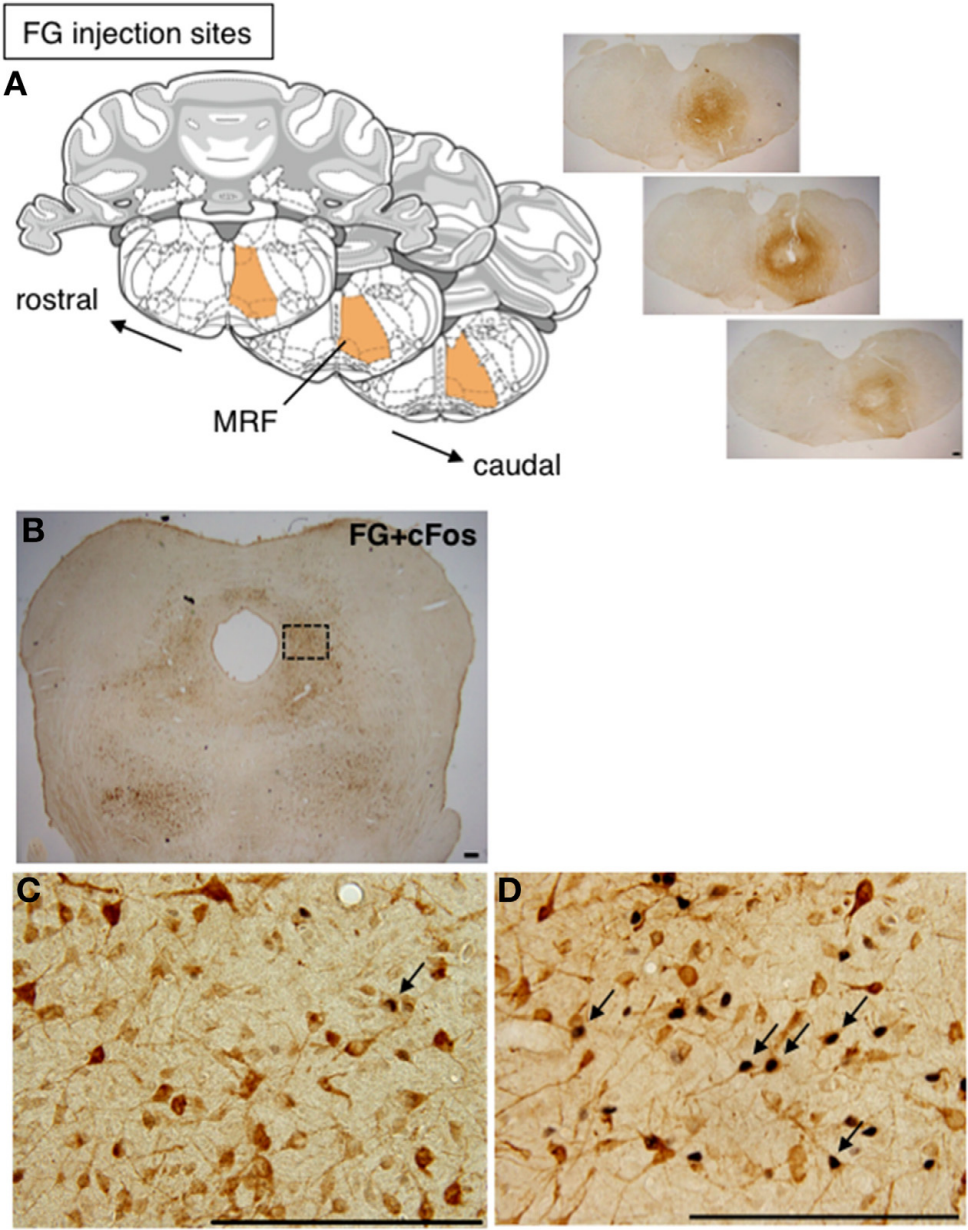
**(A)** Coronal sections from the rat brain atlas of Paxinos and Watson (2006), showing the position of the MRF and representative photomicrographs showing the site of FG injection in the RF. **(B)** Representative photomicrographs of the caudal part of the lateral PAG in rat after injection of FG into the RF. High magnification of FG-ir (brown) and cFos-ir (black) neurons in the caudal part of the lateral PAG in a non-stimulated control rat **(C)** and a mating-stimulated rat **(D)**. Arrows show FG-ir neurons with cFos-ir in nuclei. Scale bar: 200 μm.

To investigate whether cFos-expressing lateral PAG neurons induced by a mating stimulus project to the MRF, FG was injected into the MRF of female rats prior to mating stimulus. The injection of FG extended through the reticular formation (RF), including the MRF, and caudal pontine RF (PRF), with a longitudinal distance from 11.28 to 12.48 mm posterior to the bregma in the brain atlas (Paxinos and Watson, 2006) (Figure 2A). This area included the gigantocellular reticular nucleus (Gi), the gigantocellular reticular nucleus ventral (GiV) and alpha (GiA) regions, and the lateral paragigantocellular nucleus, in which neurons found neuroanatomically (Daniels et al., 1999) and electrophysiologically (Sakuma and Pfaff, 1980) have been suggested to be involved in lordosis. Many FG-ir neurons were distributed bilaterally with an ipsilateral dominance through the rostral to caudal regions of the lateral PAG (Figure 2B). In non-stimulated rats, there were a few cFos-ir and FG and cFos double-ir neurons in the lateral PAG (Figure 2C). In contrast, many FG and cFos double-ir neurons were found in the lateral PAG in mating-stimulated rats (Figure 2D). The numbers of FG and cFos-ir, cFos-ir, and FG-ir neurons in the ROI (0.5 × 0.5 mm) in the lateral PAG were 114.3 ± 7.9, 229.3 ± 19.7, and 644.5 ± 22.2, respectively. The percentage of FG-ir neurons among total cFos-ir neurons was 50.4 ± 1.7% and that of cFos-ir neurons among total FG-ir neurons was 17.9 ± 1.5% (Table 1). These numbers and percentages were significantly higher (*P* < 0.05) in mating-stimulated rats than in non-stimulated rats, except for FG-ir neurons (Table 1).

**Table 1:** **Numbers and percentages of FG and cFos immunoreactive neurons in the lateral PAG in female rats after FG injection into the RF with or without mating stimulus**.

	**Number of neurons**			**Percentages (%)**	
	**FG and cFos**	**cFos**	**FG**	**FG/cFos**	**cFos/FG**
Non-stimulus	31.0 ± 3.0	71.7 ± 8.3	709.7 ± 41.0	43.5 ± 1.5	4.4 ± 1.5
Mating-stimulus	114.3 ± 7.9^*^	229.3 ± 19.7^*^	644.5 ± 22.2	50.4 ± 1.7^*^	17.9 ± 1.5^*^

* *P < 0.05 compared with the non-stimulated rats*.

### Neurochemical identity of mating-stimulated cFos-expressing neurons in the lateral PAG

**Figure 3:**
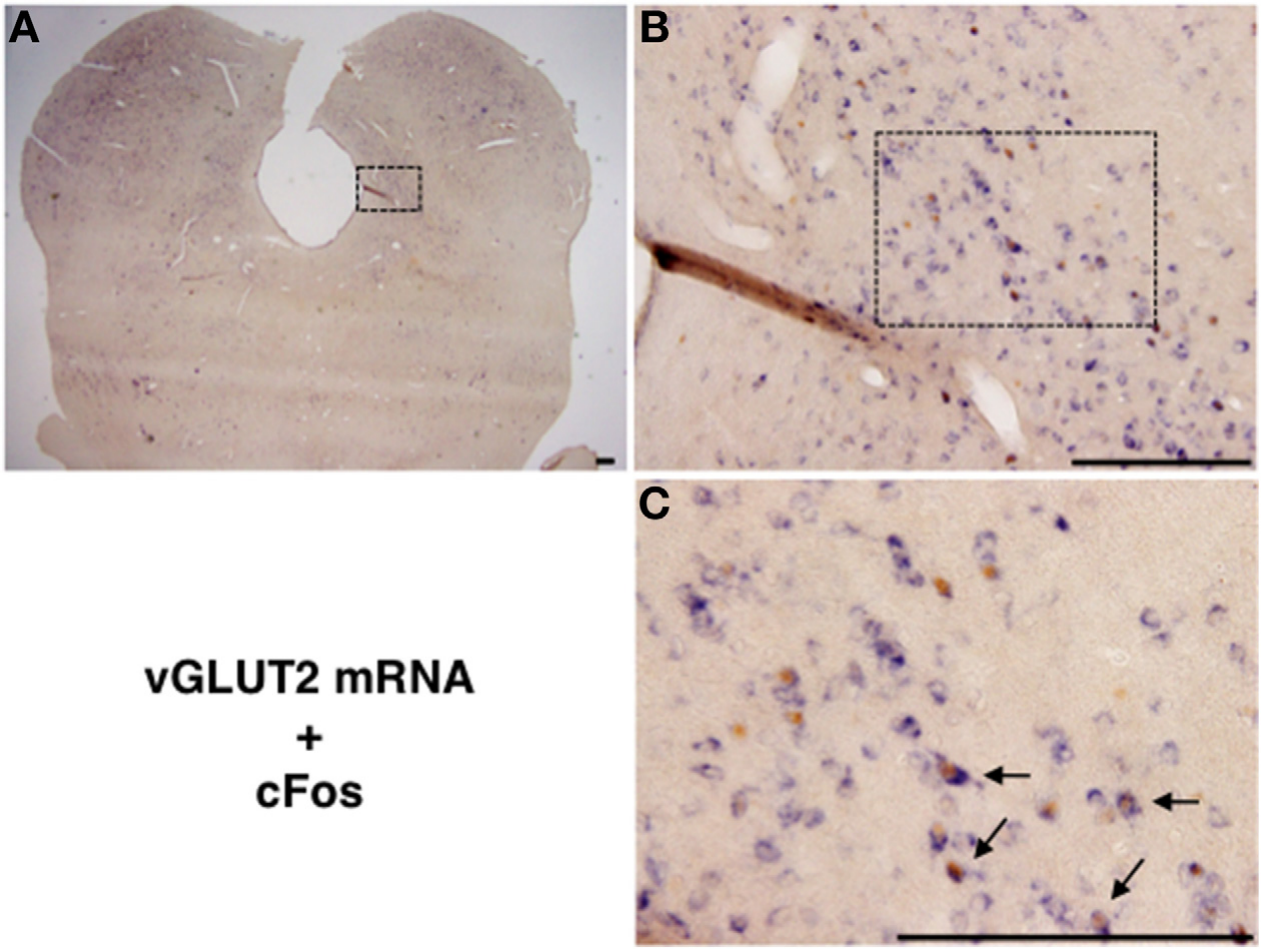
**(A)** Representative photomicrographs showing vGLUT2 mRNA (purple) *in situ* hybridization and cFos (brown) immunohistochemistry in the caudal part of the lateral PAG. **(B)** High magnification of the square in **(A)**. **(C)** Enlarged view of several neurons in the square in **(B)**. Arrows show vGLUT2 mRNA and Fos-ir neurons. Scale bar: 200 μm.

We performed double staining for *vGLUT2* mRNA ISH and cFos IHC to examine whether the mating stimulus-induced cFos-expressing neurons in the lateral PAG are glutamatergic. Many *vGLUT2* mRNA-positive neurons were located in the lateral PAG (Figures 3A,B). The distribution pattern of *vGLUT2* mRNA-positive neurons was similar to that in a previous study using another type of *vGLUT2* cRNA probe (Oka et al., 2008). Several *vGLUT2* mRNA-positive neurons showed cFos-ir in nuclei (Figure 3C). The number of *vGLUT2* mRNA-positive and cFos-ir neurons, cFos-ir neurons, and *vGLUT2* mRNA-positive neurons in the ROI (0.5 × 0.5 mm) in the lateral PAG were 74.8 ± 11.1, 131.0 ± 11.5, and 421.2 ± 31.0, respectively. The percentage of *vGLUT2* mRNA-positive neurons among total cFos-ir neurons was 55.6 ± 3.9% and that of cFos-ir neurons among total *vGLUT2* mRNA-positive neurons was 17.4 ± 1.6% (Table 2). There were no hybridization signals in brain sections incubated with sense probes for *vGLUT2* (data not shown).

**Table 2:** **Numbers and percentages of vGLUT2 mRNA-positive and cFos immunoreactive neurons in the lateral PAG in female rats after mating stimulus**.

**Number of neurons**			**Percentages (%)**	
**vGLUT2 and cFos**	**cFos**	**vGLUT2**	**vGLUT2/cFos**	**cFos/vGLUT2**
74.8 ± 11.1	131.0 ± 11.5	421.2 ± 31.0	55.6 ± 3.9	17.4 ± 1.6

*N = 6; 8 sections in each rats*.

### Projection of *vGLUT2*-positive neurons in the lateral PAG to the MRF

**Figure 4:**
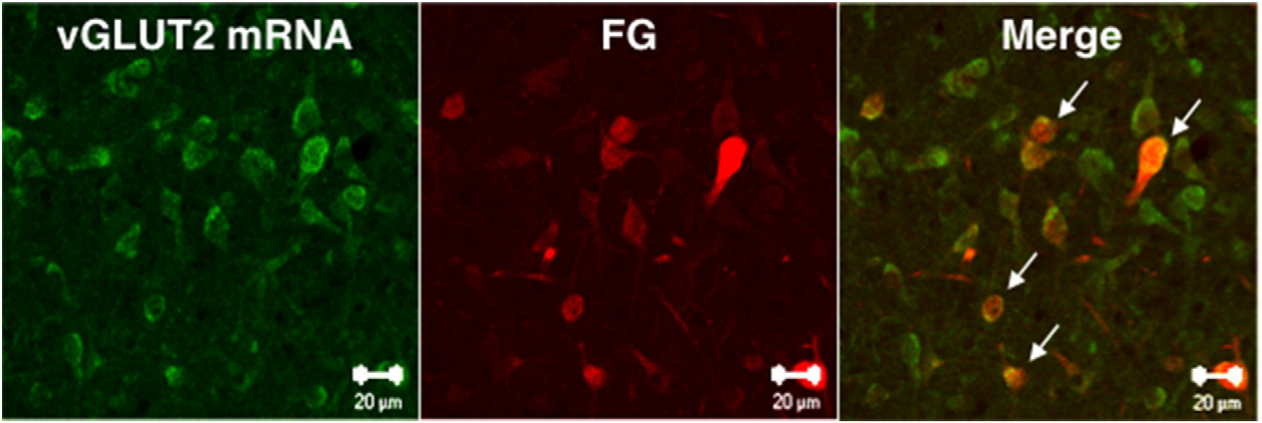
**Confocal microscope images showing vGLUT2 mRNA-positive (green) and FG-ir (red) neurons in the caudal part of lateral PAG**. Arrows indicate vGLUT2 mRNA-positive and FG-ir double-stained neurons. Scale bars: 20 μm.

We investigated whether MRF-projecting lateral PAG neurons were positive for *vGLUT2* mRNA using double fluorescence staining for *vGLUT2* mRNA ISH and FG IHC with enhancement of ISH signals by biotin-tyramide. Two out of 3 rats received a successful FG injection into the RF. Among FG-ir neurons, 75% were positive for *vGLUT2* mRNA in the lateral PAG ipsilateral to the injection site (Figure 4).

## Discussion

The results of the study show that (1) a mating stimulus activates neurons in the lateral PAG, (2) 50% of lateral PAG neurons activated by the mating stimulus project to the RF, (3) 56% of these neurons are glutamatergic, and (4) there are glutamatergic neurons projecting to the RF. The PAG and MRF are essential sites for lordosis behavior in female rats (Pfaff, 1980) and there is a clear relationship between glutamate and induction of lordosis (Gargiulo et al., 1992; Gargiulo and Donoso, 1995; Landa et al., 2009). Our findings provide further evidence that glutamatergic neurons in the lateral PAG project to the MRF and are involved in lordosis in female rats.

The PAG receives input from many brain areas, including the forebrain, hypothalamus, and brainstem, and has reciprocal efferent neurons linked with these brain areas (Paxinos, 2004). Functionally, the PAG is associated with modulation of pain and defensive behavior, in addition to lordosis (Paxinos, 2004). Thus, although we and others have shown increased cFos expression by VCS of mating or manual probing in the PAG (Tetel et al., 1993; Pfaus et al., 1996), the function of the activated PAG neurons is still not understood. In the current study, half of the mating-induced cFos-expressing neurons in the lateral PAG were found to project to the RF (MRF and PRF) using a retrograde tracer, and the MRF has also been implicated in induction of lordosis. For example, lesions in the MRF disrupt lordosis (Zemlan et al., 1983) and electrical stimulation of the MRF causes an EMG response in lordosis-inducing muscles (Femano et al., 1984). Moreover, PAG neurons are antidromically activated by electrical stimulation of the MRF in female rats (Sakuma and Pfaff, 1980). These results suggest that sensory information induced by a mount stimulus has afferent inputs in the lateral PAG and activates neurons projecting to the MRF, after which MRF neurons cause lordosis behavior in female rats.

Contradictory effects of glutamate on lordosis have been found. Icv injection of NMDA accelerated lordosis in low estrogen-primed OVX rats that rarely showed lordosis (Gargiulo and Donoso, 1995; Landa et al., 2009); whereas local injection of NMDA into the VMH inhibited lordosis in estrogen- and progesterone-treated OVX rats that showed frequent lordosis behavior (Georgescu and Pfaus, 2006). In similar rats, subcutaneous injection of MK-801, a NMDA antagonist, inhibited lordosis (Fleischmann et al., 1991), but injection into the ventral tegmental area increased lordosis (Petralia et al., 2007). In another study, an increase in lordosis induced by progesterone and luteinizing hormone-releasing hormone (LHRH) was inhibited by icv administration of a NMDA antagonist (Gargiulo et al., 1992). These results suggest condition- or region-specific effects of glutamate on lordosis behavior.

In the current study, using *vGLUT2 in situ* hybridization, we first showed activation of lateral PAG glutamatergic neurons by mating stimulus in OVX + E2 rats, indicating involvement of PAG glutamatergic neurons in lordosis. Double fluorescence for *vGLUT2* mRNA ISH and FG IHC showed the presence of lateral PAG glutamatergic neurons projecting to the RF. mRNAs for the NMDA receptor and its subunit are abundant in the MRF (Keifer and Carr, 2000; Matsuda et al., 2002). It is also likely that the NMDA receptor in the MRF is involved in lordosis because activation of lordosis-relevant muscles by electrical stimulation of the MRF was more effective in rats with additional NMDA in the MRF, compared with controls (Robbins et al., 1992). Triple-labeled histological analysis for cFos, FG IHC, and *vGLUT2* mRNA ISH was not performed, but we suggest that lateral PAG glutamatergic neurons with axonal connections to the MRF are an important neural pathway for induction of lordosis.

Several lines of evidence suggest that many neurotransmitters are related to regulation of lordosis. Thus, microinfusion of LHRH (Sakuma and Pfaff, 1983), prolactin (Harlan et al., 1983), and substance P (Dornan et al., 1987) into the PAG induces lordosis. Findings for immunoreactive nerve terminals in the PAG (Ljungdahl et al., 1978; Liposits and Setalo, 1980; Harlan et al., 1983) suggest that these peptides may be neurotransmitters or neuromodulaters that convey a mount stimulus from the spinal cord or estrogen information from the VMH to PAG glutamatergic neurons to induce lordosis.

In this study, half of mating-induced cFos-expressing neurons were not FG-ir in the lateral PAG. Some hypothalamic nuclei have an increased number of cFos-expressing neurons after mating stimulus, but not following a manual sensory stimulus of the flank and rump (Pfaus et al., 1993). Sensory stimulation of the flank and rump by a male forefoot during mount behavior is important for lordosis in female rats because denervation of the perineum, tail base, posterior rump and ventral flanks suppresses lordosis (Kow, 1976). The mating stimulus from male rats in the current study included sensory stimuli of the flank and rump and VCS in females, which suggests that the stimulus also induces activation of lateral PAG neurons that are not associated with lordosis. There are afferent projections from the PAG to the thalamus and parabrachial nucleus, which are related to cognition and pain (Sim and Joseph, 1992; Krout et al., 1998). Thus, FG-negative cFos-expressing neurons in the lateral PAG may have a role in modulating the nociceptive mechanism during lordosis. In addition, half of mating-induced cFos-expressing neurons were not glutamatergic in the lateral PAG. GABA- or neurotensin-expressing neural cell bodies are present in the PAG (Paxinos, 2004) and GABA is involved in lordosis behavior (Wada et al., 2008). This indicates that GABA neurons are a candidate for the neurons activated by mating stimulus in the lateral PAG.

We previously showed cFos IHC following ISH using a *Kiss1* DIG-labeling probe (Adachi et al., 2007). In the current study, cFos IHC following *vGLUT2* mRNA ISH was performed using the same technique, except for the difference in the DIG-labeling probe. However, cFos expression in the combination of cFos IHC with *vGLUT2* ISH was lower than that in cFos and FG double-IHC. This may have occurred because incubation with the *vGLUT2* DIG-labeling probe at 55°C might have masked an antigenic determinant of cFos for our antibody.

The precise activated area of the MRF in lordosis is not completely clear. We investigated mating stimulus-induced cFos expression in the MRF, but did not detect a cFos signal in this procedure (data not shown). Immediate early genes, including cFos, are sometimes not induced in brain regions containing neurons with spontaneous and high baseline firing rates prior to stimulation of areas such as the MRF (Pfaus and Heeb, 1997). In the current study, the widespread distribution of FG in the RF indicates the presence of mating stimulus-activated glutamatergic neurons in the lateral PAG projecting to the MRF. Our data raise the possibility that MRF neurons distributed around glutamatergic terminals from the lateral PAG can influence lordosis. Further studies are needed to address the glutamatergic influences on these neurons in regulation of lordosis.

### Conflict of interest statement

The authors declare that the research was conducted in the absence of any commercial or financial relationships that could be construed as a potential conflict of interest.
